# A negative emotional state impairs individuals’ ability to filter distractors from working memory: an ERP study

**DOI:** 10.3758/s13415-024-01166-z

**Published:** 2024-02-13

**Authors:** Chaoxiong Ye, Ruyi Liu, Lijing Guo, Guoying Zhao, Qiang Liu

**Affiliations:** 1 Institute of Brain and Psychological Sciences, Sichuan Normal University, 610068 Chengdu, China; 2 Department of Psychology, University of Jyvaskyla, 40014 Jyvaskyla, Finland; 3 Center for Machine Vision and Signal Analysis, University of Oulu, 90014 Oulu, Finland; 4 Research Center of Brain and Cognitive Neuroscience, Liaoning Normal University, 116029 Dalian, China

**Keywords:** Negative emotion, Contralateral delay activity, Distractor filtering, Visual short-term memory

## Abstract

Capacity-limited visual working memory (VWM) requires that individuals have sufficient memory space and the ability to filter distractors. Negative emotional states are known to impact VWM storage, yet their influence on distractor filtering within VWM remains underexplored. We conducted direct neural measurement of participants (n = 56) who conducted a lateralized change detection task with distractors, while manipulating the emotional state by presenting neutral or negative images before each trial. We found a detrimental effect of distractors on memory accuracy under both neutral and negative emotional states. Using the event-related potential (ERP) component, contralateral delay activity (CDA; sensitive to VWM load), to observe the VWM load in each condition, we found that in the neutral state, the participants showed significantly higher late CDA amplitudes when remembering 4 targets compared with 2 targets and 2 targets with 2 distractors but no significant difference when remembering 2 targets compared with 2 targets with 2 distractors. In the negative state, no significant CDA amplitude differences were evident when remembering 4 targets and 2 targets, but CDA was significantly higher when remembering 2 targets with 2 distractors compared with 2 targets. These results suggest that the maximum number of items participants could store in VWM was lower under negative emotional states than under neutral emotional states. Importantly, the participants could filter out distractors when in a neutral emotional state but not in a negative emotional state, indicating that negative emotional states impair their ability to filter out distractors in VWM.

**Supplementary Information:**

The online version contains supplementary material available at 10.3758/s13415-024-01166-z.

## Introduction

Visual working memory (VWM) refers to the cognitive system responsible for temporarily storing and manipulating visual information. It enables the retention of visual stimuli in the mind, even after they are no longer present in the environment. VWM predicts individual differences in fluid intelligence (Burgess et al., 2011; Fukuda et al., 2010; Unsworth et al., 2014) and performance on general cognitive tasks (Dehn, 2017; Unsworth et al., 2015). However, the capacity of VWM is limited, and its accuracy decreases when attempting to maintain more than three or four simple items (Luck & Vogel, 1997, 2013; Vogel et al., 2001). The visual system often faces task demands that exceed the limits of VWM; therefore, selective regulation of access to task-relevant stimuli in VWM and filtering out task-irrelevant distractors are crucial. Consequently, substantial literature has emerged on the topic of distractor filtering in VWM (Allon & Luria, 2019; Feldmann-Wustefeld & Vogel, 2019; Maniglia & Souza, 2020; Plebanek & Sloutsky, 2019).

The direct observation of brain activity and the high temporal resolution advantages offered by electroencephalogram (EEG) technology have allowed researchers to use event-related potential (ERP) measurements to investigate VWM storage and distractor filtering in VWM (Vogel & Machizawa, 2004; Vogel et al., 2005). One frequently studied ERP component in VWM studies is contralateral delay activity (CDA), a sustained negative potential that reflects the information currently held in VWM. Previous research has extensively utilized CDA to examine VWM processes (Figueira et al., 2017, 2018; Ikkai et al., 2010; Xie & Zhang, 2018; Xu et al., 2018). In general, the amplitude of CDA increases as the number of representations in VWM increases; however, once individuals reach the upper limit of their VWM capacities, the CDA amplitude no longer increases with the number of items to be remembered (Feldmann-Wustefeld et al., 2018; Vogel & Machizawa, 2004). Consequently, the CDA amplitude can serve as an indicator of the number of items stored in VWM by individuals and provide insights into the allocation of VWM resources to the stored representations.

In a pioneering study conducted by Vogel et al. (2005), the researchers utilized the CDA component to explore how distractors were filtered in VWM. The experiment involved having participants memorize the orientations of red rectangles, occasionally in the presence of blue rectangles that served as task-irrelevant distractors. Participants with high VWM capacity exhibited indistinguishable CDA amplitudes when remembering two red items with two blue distractors or two red items alone. This finding suggests that participants with high VWM capacity efficiently focused on representing the relevant red items while disregarding the irrelevant ones in VWM. Conversely, participants with low VWM capacity displayed comparable CDA amplitudes, whether remembering two red items with distractors or remembering four red items alone. This indicates that participants with low VWM capacity struggled to exclude irrelevant items from their VWM. Consequently, the CDA in this experimental paradigm can be regarded as a neurophysiological marker for determining the quantity of relevant and irrelevant items retained in VWM during a task’s retention interval.

Previous studies utilizing EEG technology and the CDA component have identified various factors that influence distractor filtering in VWM. For instance, Jost et al. (2011) found that distractor filtering was slower in older adults than in younger individuals with low VWM capacity. Lee et al. (2010) discovered impaired distractor filtering in patients with Parkinson’s disease relative to healthy controls. Xu et al. (2018) observed a decline in distractor filtering abilities following social exclusion. Owens et al. (2012) demonstrated lower distractor filtering abilities in individuals with depression symptoms than in nondepressed individuals. Song et al. (2021) found weaker distractor filtering abilities in individuals with high test anxiety than with low test anxiety. Qi et al. (2014) revealed lower distractor filtering abilities in individuals with high trait anxiety than with low trait anxiety. However, Ward et al. (2020) found that shock-induced state anxiety, although it reduces participants’ VWM capacity, does not affect their distractor filtering abilities. Spronk et al. (2013) also found no significant differences in distractor filtering abilities between adolescents and adults with attention-deficit/hyperactivity disorder. Furthermore, in studies manipulating experimental stimuli, researchers found that both healthy individuals and those with high trait anxiety struggle to filter out fearful face distractors from VWM (Stout et al., 2013; Ye et al., 2023), while individuals with low VWM capacity have difficulty filtering potentially threatening face distractors (Ye et al., 2018). Collectively, these studies indicate that distractor filtering abilities in VWM processing are influenced by various factors, particularly individual abilities and traits.

The memory process is also widely acknowledged to be influenced by individuals' temporary emotional states, such as negative emotional states. Negative emotional states can yield certain benefits to memory, such as the formation of flashbulb memories (Brown & Kulik, 1977), because negative emotions enhance the subjective vividness of a memory while also increasing the likelihood of remembering some event details while forgetting others (see a review by Kensinger, 2007). Recently, many studies have investigated the influence of emotional states on VWM processing. For example, the behavioral experiment by Xie and Zhang (2016), which examined the impact of different emotional states on VWM storage, revealed no effect of positive emotions on VWM processing but enhanced memory precision of VWM representations in response to negative emotions. Furthermore, Xie et al. (2022) discovered that negative emotional states accelerate the consolidation speed of VWM representations. Spachtholz et al. (2014) found that an improvement in VWM precision due to negative emotions may come at the cost of sacrificing the overall quantity of memory. This cost is now supported by ERP evidence confirming that negative emotional states may reduce the upper limit of VWM capacity in individuals (Figueira et al., 2017). In our recent study, we also found that negative emotional states influence VWM in the late phase of resource allocation, and that a trade-off occurs between VWM precision enhancement and VWM quantity reduction (Long et al., 2020). Moreover, our recent meta-analysis, based on data from 13 experiments, involving 491 participants performing a delay-estimation VWM task under negative and neutral emotional states, showed that induced negative emotional states moderately decreased VWM recall variability (i.e., negative emotional states improved VWM precision) and increased recall failures compared with the neutral condition (Xie et al., 2023). A comprehensive understanding has emerged regarding the effects of negative emotional states on VWM storage; however, to the best of our knowledge, little previous ERP research has investigated the impact of negative emotional states on distractor filtering abilities in VWM processing.

The purpose of the present study was to address this gap in knowledge by investigating the impact of negative emotional states on the ability to filter out distractors in VWM. Our emotional state manipulation followed a method similar to that used in our previous research (Long et al., 2020), wherein participants were presented with neutral or negative images at the beginning of each trial within different emotional state blocks. We assessed participants’ distractor filtering ability for neutral stimuli using the CDA component by having the participants perform a lateralized change detection task similar to that used by Vogel et al. (2005), while EEG signals were recorded. We anticipated that negative emotional states might improve participants’ VWM precision but at the expense of VWM capacity. As CDA primarily reflects the number of items stored in VWM, we expected our CDA analysis to show a reduction in the maximum number of items the participants could store in VWM during negative emotional states. Specifically, we expected that participants in a neutral emotional state would show significantly higher CDA amplitudes for high load conditions compared with low memory load conditions, but no significant difference in CDA amplitudes between these conditions in negative emotional states. Furthermore, two possible effects of negative emotional states on distractor filtering ability were considered. One possibility was that negative emotional states, similar to their positive influence on VWM storage, also might enhance the distractor filtering ability. Consequently, participants would effectively filter out distractors during negative emotional states, as reflected by comparable CDA amplitudes between distractor conditions and low memory load conditions. The other possibility was that negative emotional states would have a different effect on distractor filtering than on VWM storage. Negative emotional states might weaken the participants’ ability to filter out distractors, thereby increasing the unnecessary storage of distractors in VWM during blocks with negative emotional states. This would manifest as significantly higher CDA amplitudes in distractor conditions than in low memory load conditions under negative emotional states. Therefore, examining the influence of negative emotional states on VWM distractor filtering was a prime objective. Moreover, we also considered that individuals might experience different phases in VWM consolidation and storage (Long et al., 2020; Qi et al., 2014; Xie & Zhang, 2018; Ye et al., 2017, 2019, 2020). For instance, our previously proposed two-phase VWM resource allocation model suggests that VWM consolidation can be distinguished into early and late phases. During the early phase, resources are involuntarily allocated to all visual stimuli, forming as many low-resolution representations as possible. In the later phase, individuals can voluntarily reallocate VWM resources according to task demands, focusing more resources on target items to refine their representations (Ye et al., 2017). Based on this model, our previous study also indicated that the impact of negative emotional states on VWM storage primarily occurs during the late VWM consolidation phase, without affecting the early phase (Long et al., 2020). Therefore, our analysis explored the overall impact of emotional states on VWM filtering, as well as the effects of these emotional states on distractor filtering during different VWM maintenance phases (early or late). The findings could provide valuable insights into how emotions modulate cognitive processes and may have potential implications for understanding emotional regulation and its impact on attentional control.

## Methods

### Power analysis

The current 2 (neutral emotional state vs. negative emotional state) × 3 (two targets vs. two targets with two distractors vs. four targets) within-subjects design for repeated-measures analysis of variances (ANOVA) was subjected to a power analysis. Repeated-measures ANOVA was employed as our primary analytical approach, with the goal of examining the interaction between emotional state and memory condition on the CDA. We anticipated a moderate-to-small effect size (e.g., η_p_^2^ = 0.10) with a statistical power of 85% at a significance level of 0.05 (Faul et al., 2007). The power analysis indicated a minimum sample size of 54 participants.

### Participants

A group of 72 college participants from Liaoning Normal University willingly took part in the study and received monetary compensation. The inclusion criteria were aged 17 years or older, self-reported normal color vision, and normal or corrected-to-normal visual acuity. Exclusion criteria included a history of psychiatric disorders, use of nervous system acting drugs, and previous participation in working memory experiments. Sixteen participants were excluded because of extensive electroencephalogram artifacts and eye movements, resulting in a final sample of 56 participants (mean age 22.14 ± 3.05 years [mean ± standard deviation], age range 17–40 years; 4 left-handed; 29 females and 27 males) for subsequent data analysis. This chosen sample size aligns with previous research that employed a similar experimental paradigm (60 participants for Ward et al., 2020). Before the experiment, written, informed consent was obtained from each participant. All procedures adhered to the guidelines stated in the Declaration of Helsinki and were approved by the ethics committees of Liaoning Normal University and Sichuan Normal University.

### Materials

The entire experiment was conducted using E-Prime 2.0 (Psychology Software Tools, Inc.). The stimuli were presented on an LCD monitor with a gray background (6.1 cd/m^2^, RGB: 128, 128, 128) at a viewing distance of 70 cm. Similar to previous research on the induction of negative emotional states (Figueira et al., 2017; Long et al., 2020; Xie & Zhang, 2016, 2017), we manipulated the participants' emotional states by presenting them with images before each trial. These images consisted of 60 neutral black-and-white images and 60 negative black-and-white images, consistent with the emotion-inducing images used in our recent studies (Long et al., 2020). These images were originally selected from the International Affective Picture System (IAPS) database (Lang & Bradley, 2007). Based on the assessment by Lang & Bradley (2007), each selected image demonstrated a high level of agreement in terms of emotion categorization. A *t*-test analysis revealed a significant difference in valence between the negative (2.45 ± 0.73) and neutral (5.51 ± 0.66) images, t (118) = 24.02, *p* < 0.001. Arousal levels were also assessed for the negative (5.57 ± 0.80) and neutral (3.32 ± 0.80) images, and the *t*-test analysis showed a significant difference as well, t (118) = 16.62, *p* < 0.001.

In each trial, the participants were presented with a corresponding emotional image prior to completing a lateralized change detection task within a neutral or negative emotional block. The memory arrays were displayed within two invisible rectangular regions, each measuring 5.73° × 6.55°, and positioned 4° to the left and right of a central fixation cross. The memory arrays consisted of two or four colored rectangles (0.65° × 1.20°) with different orientations, presented separately in each hemifield. A minimum of two visual angles separated each stimulus. These rectangles were filled with two colors—red (RGB: 255, 0, 0) and blue (RGB: 0, 32, 96)—and had four orientations (vertical, horizontal, left 45°, and right 45°). The memory array encompassed three conditions: two target rectangles in each hemifield (referred to as the 2T condition), two target rectangles with two distractor rectangles in each hemifield (the 2T2D condition), and four target rectangles in each hemifield (the 4T condition). This experimental setup is consistent with those used in previous VWM studies involving distractor filtering tasks (Owens et al., 2012; Vogel et al., 2005).

### Procedure

#### EEG experiment

**Fig. 1:**
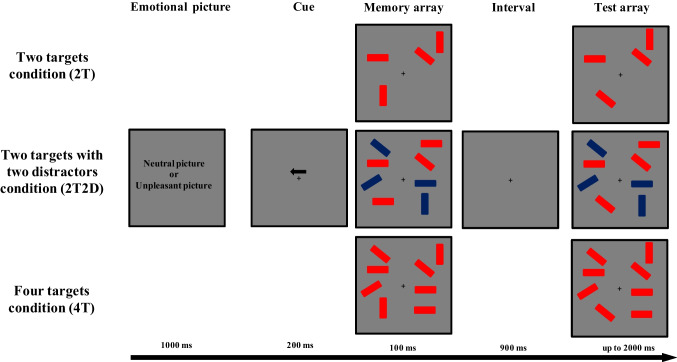
Flowchart of the main task. A neutral or negative image was presented before each trial in the corresponding emotional state block of the lateralized change-detection task. The sequence of emotional state blocks was counterbalanced across the participants. The flowchart depicts the scenario with red targets and blue distractors. The participants were instructed to determine whether the orientations of the red rectangles (targets) changed in the cued hemifield. The cued hemifield was the left hemifield in half of the trials (as shown in the flowchart), and the right hemifield in the remaining trials. The participants were instructed to ignore the blue rectangles (distractors). The memory condition varied with a two-target array (2T), a two-target array with two distractors (2T2D), and a four-target array (4T)

For the EEG experiment, the participants completed a VWM task with two emotional blocks: a neutral emotional state block and a negative emotional state block. The order of block execution was counterbalanced across the participants. The flowchart for each trial is depicted in Fig. 1. During each trial, participants viewed either a neutral picture (during the neutral emotional block) or a negative picture (during the negative emotional block). The duration of the picture presentation was set to 1000 ms. An important point to note is that these pictures were unrelated to the experimental task. The participants were not required to respond to the presented neutral or negative pictures. An arrow cue appeared 600 to 700 ms after the pictures disappeared. The arrow, displayed above the fixation cross for 200 ms, indicated the hemifield (left or right) to which the participants should attend. In 50% of the trials, the arrow pointed to the left, while in the remaining trials, it pointed to the right. Following an interval of 200 to 400 ms, the memory array was presented for 100 ms. Participants were instructed to memorize the orientations of the target rectangles while disregarding the distractor rectangles in the cued hemifield of the memory array. The color of the target rectangles (red or blue) was counterbalanced across the participants. After a blank retention period of 900 ms, the test array appeared. The participants were required to indicate, by pressing a key, whether the direction of the target rectangles had changed. In half of the trials, one of the targets changed, while in the other half, nothing changed. Emphasis was placed on accuracy rather than response speed. The participants were instructed to maintain fixation and refrain from moving their heads throughout the entire trial.

At the onset of the experiment, the participants were required to complete the negative affect section of the Positive and Negative Affect Schedule (PANAS, Watson et al., 1988) to assess their emotional state. This allowed us to gather information about the participants' emotional state before the EEG experiment. Following completion of the emotional state questionnaire, the participants engaged in practice trials designed to mirror the formal experiment. The aim of these practice trials was to familiarize the participants with the task and ensure that they understood the procedures. Before each formal emotional state block, the participants needed to perform at least 12 practice trials, during which they received feedback on the accuracy of their responses after each trial. However, during the formal experiment, no feedback was provided regarding participant responses in each trial.

After completing each emotional state block, the participants were again required to complete the negative affect section of the PANAS to measure their emotional state. In total, the participants completed the negative affect questionnaire three times throughout the entire experiment: before the first emotional block, after the first emotional block, but before the second block, and after the second emotional block.

Within both the neutral emotional state block and the negative emotional state block, there were 360 trials. These trials were equally distributed among three memory conditions: two targets (2T), two targets with two distractors (2T2D), and four targets (4T). The assignment of the cued hemifield and memory condition was randomized within each emotional state to ensure unbiased results. Each participant completed a total of 720 trials in this task.

A brief interval of 3 to 5 min was inserted between emotional blocks to provide participants with a short break and allow them to transition between emotional states. Additionally, two mini-breaks of at least 15 s were provided within each block to minimize fatigue and maintain participant engagement. Overall, the duration of the EEG experiment was approximately 1 hr.

#### Pre-experiment procedures

Before commencing the EEG experiment, comprehensive assessments were conducted to gather information on the participants’ anxiety symptoms, depression symptoms, and VWM capacity. The participants completed standardized questionnaires, including the State-Trait Anxiety Inventory (STAI, Spielberger et al., 1971) and the Beck Depression Inventory II (BDI-II, Beck et al., 1996) to evaluate their anxiety and depression levels. Following questionnaire completion, the participants participated in a behavioral experiment known as the change detection task, designed to measure their VWM capacity (for detailed experimental procedures, please see the Supplementary Materials). The initial assessment phase lasted approximately 20 min. Subsequently, the participants had a resting period in the laboratory, lasting approximately 30 min to 1 hr, to prepare for the EEG experiment. Upon conclusion of the preparation phase, the participants proceeded with the EEG experiment.

#### Electroencephalogram recording and analysis

During the task, we continuously recorded electroencephalogram (EEG) activity using a 64-channel active Ag/AgCl electrode system (Brain Products ACTi Champ) positioned on an elastic cap, according to the International 10-10 system. The ground electrode was placed at FPz. The online reference for the data was set to the vertex (Cz). For the post-recording analyses, the data were re-referenced offline to the average of the bilateral mastoids. A vertical electrooculogram (VEOG) was recorded using a bipolar-referenced electrode pair, with one electrode placed above and the other below the right eye. A horizontal electrooculogram (HEOG) was recorded by using a bipolar-referenced electrode pair positioned approximately 1 cm laterally to the outer canthi of both eyes. The impedance at each electrode site was kept below 5 kΩ. The EEG and EOG signals were digitized at a sampling rate of 500 Hz.

The EEG data of the participant with missing behavioral data were intact; consequently, we retained these EEG data for subsequent analysis. The data were processed offline by using BrainVision Analyzer 2.1 (Brain Products GmbH, Munich, Germany). The EEG signals were segmented into epochs of 1,200-ms duration, starting from 200 ms before the onset of the memory array. A low-pass filter with a cutoff frequency of 30 Hz was applied to the data. Baseline correction was performed by subtracting the average amplitude of the 200-ms prestimulus interval. Trials containing horizontal eye movements, identified by HEOG amplitudes exceeding ±32 μV, were excluded from the analysis. Additionally, trials with remaining artifacts exceeding ±75 μV in amplitude were rejected. Participants with a trial rejection rate higher than 30% were excluded from further analysis. The EEG data from the remaining trials were averaged for each participant and condition, and the averages were time-locked to the onset of the memory array.

Following the protocol used in previous research (Feldmann-Wustefeld & Vogel, 2019), we selected one pair of posterior electrode sites (PO7/PO8) for our analysis. In each block and for each stimulus condition, the contralateral waveforms were calculated for each participant by averaging the activity recorded at the left hemisphere electrode sites when the participants were cued to memorize the right side of the memory array. For the opposite condition, the activity recorded at the right hemisphere electrode sites was averaged when participants were cued to memorize the left side. The ipsilateral waveforms were computed by averaging the activity from both the left and right hemisphere sites when participants were cued to memorize the left and right sides of the memory array, respectively. The whole CDA amplitude was determined by subtracting the ipsilateral activity from the contralateral activity within a measurement window of 300–1000 ms after the onset of the memory array.

Based on previous research suggesting differences between early and late CDA components reflecting various VWM storage phases (Qi et al., 2014; Xie & Zhang, 2018), we analyzed two equal-length time windows for CDA: early (300–600 ms) and late (700–1000 ms). This allowed us to obtain reliable statistical comparisons of CDA amplitudes. We decomposed these CDA amplitudes (early and late) separately for each experimental condition and participant. Our primary ERP measures included the whole CDA amplitude (300–1000 ms), early CDA amplitude (300–600 ms), and late CDA amplitude (700–1000 ms), with the latter two measures providing a more detailed representation of the overall CDA results.

In addition to the results for difference waveforms, we conducted exploratory analyses to investigate other early contralateral activities related to attention, such as the N2pc components (Eimer, 1996). For more comprehensive details of the results and discussion related to these analyses, please see the Supplementary Materials.

### Statistical analysis

We analyzed the changes in the participants’ emotional states in each block by calculating the emotional state change scores for the neutral emotional state block and the negative emotional state block. This was done by subtracting the negative affect score of the PANAS questionnaire after the neutral emotional state block from that before the neutral emotional state block, and the result reflected the impact of the neutral emotional state block on the participants’ emotional states. We also subtracted the negative affect score of the PANAS questionnaire after the negative emotional state block from that before the negative emotional state block to obtain the emotional state change scores for the negative emotional state block. The results indicated the influence of the negative emotional state block on the participants’ emotional states. Larger scores for the emotional state change indicated a stronger negative emotional state induced by the experimental block. Subsequently, we employed two-tailed, one-sample *t*-tests to examine the differences between the emotional state change scores (for the neutral or negative emotional state block) and zero. We also used a two-tailed, paired *t*-test to compare the differences between the emotional state change scores for the neutral emotional state block and the negative emotional state block.

We analyzed the behavioral accuracy and whole CDA amplitude by performing a two-way repeated measures ANOVA, with emotional state (neutral vs. negative) and memory condition (2T vs. 2T2D vs. 4T) considered as within-subject factors. We also analyzed early and late CDA amplitudes using a three-way repeated measures ANOVA, with time window (early vs. late), emotional state (neutral vs. negative), and memory condition (2T vs. 2T2D vs. 4T) considered as within-subject factors. Planned pairwise comparisons within each emotional block were conducted using two-tailed paired *t*-tests to compare differences between the 2T and 4T conditions, 2T and 2T2D conditions, and 2T2D and 4T conditions. In terms of CDA amplitude results, a significantly higher CDA amplitude for the 4T compared to 2T would indicate that participants could memorize more than two targets in full-target trials. Conversely, if the 4T and 2T conditions did not differ, this would suggest a maximum VWM capacity of the two targets. Similarly, no significant difference between the 2T and 2T2D conditions would imply that the participants did not store additional distractors in VWM, whereas a higher amplitude for 2T2D than 2T would indicate distractor storage. Additionally, a significantly higher CDA amplitude for the 4T compared with the 2T2D condition would suggest that more information was stored in the high memory load condition than in the distractor condition. A finding of no significant difference between the 4T and 2T2D conditions would imply an inability to filter distractors, whereas a higher amplitude for 2T2D than 4T would suggest the storage of more information under the distractor condition than under the high memory load condition.

The value of *η*_*p*_^*2*^ was used as an estimator of the effect size for the ANOVA. Cohen's d was used as an estimator of the effect size for the t-tests and provided an indication of the magnitude of the observed effects. A significance level of *p* < 0.05 was adopted for all statistical tests. Marginally significant results (0.05 < *p* < 0.10) also were reported to acknowledge trends that may be of interest. To avoid drawing conclusions based solely on null results that might be observed because of chance, we utilized Bayes factor analysis (Rouder et al., 2009). The Bayes factor (BF_10_) was employed to compute an odds ratio for the alternative hypothesis compared with the null hypothesis (values < 1 favor the null hypothesis, while values > 1 favor the alternative hypothesis). This approach provides a more nuanced assessment of the evidence for or against the alternative hypothesis by considering both the data and prior information. In addition, based on our assessment of the participants' VWM capacities, anxiety symptoms, depression symptoms and degree of negative induction, we did not find any significant new evidence indicating a meaningful relationship between these factors and the participants' EEG results; therefore, we have not reported those results in the main text. Additionally, as our experiment emphasized accuracy over speed, no analysis of reaction times is reported in the main text. Please see the Supplementary Materials for analyses and discussions of these results. All data mentioned in the main text and Supplementary Materials are available through the Open Science Framework at https://osf.io/bshm2/.

## Results

### Emotional state change results

The results of the negative affect scores for the PANAS questionnaire indicated that the scores for the change in emotional state were significantly lower than zero in the neutral emotional state block (−1.59 ± 4.979), t (55) = 2.389, *p* = 0.020, Cohen’s d = 0.319, BF_10_ = 1.961. This suggests that the participants reported a significant decrease in their perceived level of negative affect following the completion of the neutral emotional state block. Conversely, the scores for the change in emotional state were significantly higher than zero in the negative emotional state block (2.57 ± 6.835), t (55) = 2.815, *p* = 0.007, Cohen’s d = 0.376, BF_10_ = 5.058, indicating that the participants experienced a significant increase in their reported level of negative affect following the negative emotional state block. Furthermore, the scores for the change in emotional state were significantly higher in the negative emotional state block than in the neutral emotional state block, t (55) = 2.981, *p* = 0.004, Cohen’s d = 0.398, BF_10_ = 7.539. This result suggests a stronger negative emotional state induced by the negative emotional block than by the neutral emotional block.

### Accuracy results

**Fig. 2:**
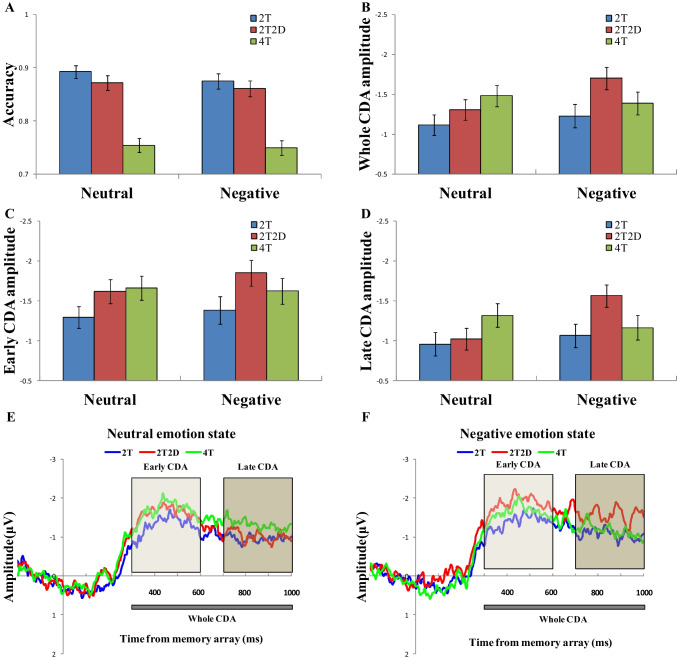
Results of each experimental condition. (**A**) Accuracy results (mean and standard error of the mean) are presented separately for the neutral emotional state (left) and negative emotional state (right) across different memory conditions. (**B**) Whole CDA amplitude (300–1000 ms) results for the neutral emotional state (left) and negative emotional state (right) are shown separately under different memory conditions. (**C**) Early CDA amplitude (300–600 ms) results for the neutral emotional state (left) and negative emotional state (right) are shown separately under different memory conditions. (**D**) Late CDA amplitude (700–1000 ms) results for the neutral emotional state (left) and negative emotional state (right) are shown separately under different memory conditions. Error bars represent the standard error. (**E**) Difference waveforms (contralateral waves minus ipsilateral waves) of average ERPs are depicted for different memory conditions in the neutral emotional state. (**F**) Difference waveforms of average ERPs are presented for different memory conditions in the negative emotional state. The waveforms are time-locked to the onset of the memory array (y-axis at time zero)

The mean accuracy for each memory condition (2T vs. 2T2D vs. 4T) under neutral or negative emotional states is presented in Fig. 2A. The analysis of variance (ANOVA) revealed a significant main effect of the memory condition (mean accuracy for the 2T, 2T2D and 4T conditions: 0.884 ± 0.0969, 0.866 ± 0.104, 0.752 ± 0.0961, respectively), F (2,110) = 196.943, *p* < 0.001, *η*_*p*_^*2*^ = 0.782. A marginally significant main effect of the emotional state also was apparent (mean accuracy for the neutral emotional state and negative emotional state: 0.840 ± 0.0907, 0.828 ± 0.103, respectively, F (1,55) = 3.260, *p* = 0.076, *η*_*p*_^*2*^ = 0.056. However, no significant interaction was evident between the memory condition and emotional state, F (2,110) = 1.220, *p* = 0.299, *η*_*p*_^*2*^ = 0.022.

### Whole CDA component (300–1000 ms) results

The averaged difference waveforms for the neutral and negative emotional states are presented in Fig. 2E and F, respectively. Figure 2B displays the whole CDA amplitudes across all memory conditions for both the neutral and negative emotional states. The ANOVA revealed a significant main effect of the memory condition, F (2,110) = 6.30, *p* = 0.003, *η*_*p*_^*2*^ = 0.103, and a marginally significant interaction between the memory condition and emotional condition, F(2,110) = 2.71, *p* = 0.071, *η*_*p*_^*2*^ = 0.047. However, no significant main effect of the emotional state was found, F (1,55) = 2.46, *p* = 0.12, *η*_*p*_^*2*^ = 0.043.

Planned pairwise comparisons revealed that in the neutral emotional state, the whole CDA amplitudes were significantly larger for the 4T condition (−1.48 ± 1.00) than for the 2T condition (−1.12 ± 0.96), t (55) = 2.45, *p* = 0.018, Cohen’s d = 0.327, BF_10_ = 2.21. Additionally, the whole CDA amplitudes showed a marginally significant difference between the 2T2D condition (−1.31 ± 0.96) and the 2T condition, t(55) = 1.76, *p* = 0.084, Cohen’s d = 0.235, BF_10_ = 0.62. However, no significant differences were observed between the 2T2D condition and the 4T condition, t(55) = 1.23, *p* = 0.22, Cohen’s d = 0.165, BF_10_ = 0.30. Conversely, in the negative emotional state, the whole CDA amplitudes were significantly higher for the 2T2D condition (−1.70 ± 1.04) than for the 2T condition (−1.23 ± 1.12), t(55) = 3.38, *p* < 0.001, Cohen’s d = 0.451, BF_10_ = 21.02, and showed a marginally significant difference compared with the 4T condition (−1.39 ± 1.07), t(55) = 1.83, *p* = 0.073, Cohen’s d = 0.245, BF_10_ = 0.69. However, no significant difference was detected in the whole CDA amplitudes between the 2T condition and the 4T condition, t(55) = 1.09, *p* = 0.281, Cohen’s d = 0.146, BF_10_ = 0.26. Furthermore, the results indicated that the whole CDA amplitudes of the 2T2D condition were significantly higher in the negative emotional state than in the neutral emotional state, t(55) = 2.632, *p* = 0.011, Cohen’s d = 0.352, BF_10_ = 3.32. However, no significant differences in CDA amplitudes were observed between the neutral and negative emotional states for the 2T condition, t(55) = 0.86, *p* = 0.393, Cohen’s d = 0.115, BF_10_ = 0.21, or between the neutral and negative emotional states for the 4T condition, t(55) = 0.547, *p* = 0.586, Cohen’s d = 0.073, BF_10_ = 0.17.

### Early CDA component (300–600 ms) and late CDA component (700–1000 ms) results

Figure 2C and D illustrate the early CDA amplitudes (300–600 ms) and late CDA amplitudes (700–1000 ms) for all memory conditions in both negative and neutral emotional blocks. The three-way repeated measures ANOVA revealed a significant interaction between the time window, memory condition, and emotional condition, F (2,110) = 3.642, *p* = 0.030, *η*_*p*_^*2*^ = 0.062, a marginally significant interaction between the memory condition and emotion condition, F (2,110) = 2.654, *p* = 0.075, *η*_*p*_^*2*^ = 0.046, a significant main effect of the time window, F (1,55) = 15.218, *p* < 0.001, *η*_*p*_^*2*^ = 0.217, and a significant main effect of the memory condition, F (2,110) = 6.663, *p* = 0.002, *η*_*p*_^*2*^ = 0.108, but no significant interaction between time windows and emotion, F (1,55) = 1.255, *p* = 0.267, *η*_*p*_^*2*^ = 0.022, no significant interaction between time windows and condition, F (1,55) = 1.145, *p* = 0.322, *η*_*p*_^*2*^ = 0.020, and no significant main effect of emotion, F (1,55) = 2.099, *p* = 0.153, *η*_*p*_^*2*^ = 0.037.

For the early CDA component, planned pairwise comparisons indicated that in the neutral emotional state, the early CDA amplitudes were significantly larger for the 2T2D condition (−1.62 ± 1.13) than for the 2T condition (−1.29 ± 1.02), t (55) = 2.848, *p* = 0.006, Cohen’s d = 0.381, BF_10_ = 5.469. Furthermore, the early CDA amplitude was significantly higher in the 4T condition (−1.66 ± 1.12) than in the 2T condition, t (55) = 2.926, *p* = 0.005, Cohen’s d = 0.392, BF_10_ = 6.597, but no significant difference was observed between the 2T2D condition and the 4T condition, t (55) = 0.401, *p* = 0.690, Cohen’s d = 0.054, BF_10_ = 0.158. In addition, during the negative emotional state, the early CDA amplitudes were significantly larger for the 2T2D condition (−1.85 ± 1.20) than for the 2T condition (−1.38 ± 1.31), t (55) = 3.762, *p* < 0.001, Cohen’s d = 0.503, BF_10_ = 61.235. However, no significant differences were found between the 2T condition and the 4T condition (−1.62 ± 1.19), t (55) = 1.594, *p* = 0.117, Cohen’s d = 0.213, BF_10_ = 0.479, or between the 2T2D and 4T conditions, t (55) = 1.451, *p* = 0.153, Cohen’s d = 0.194, BF_10_ = 0.392.

For the late CDA component, planned pairwise comparisons revealed that in the neutral emotional state, the late CDA amplitudes were marginally larger in the 4T condition (−1.32 ± 1.11) than in the 2T condition (−0.96 ± 1.08), t (55) = 1.958, *p* = 0.055, Cohen’s d = 0.262, BF_10_ = 0.859. However, no significant differences were noted in late CDA amplitudes between the 2T2D condition (−1.02 ± 1.02) and the 2T condition, t (55) = 0.491, *p* = 0.625, Cohen’s d = 0.066, BF_10_ = 0.164, or between the 2T2D condition and the 4T condition, t (55) = 1.539, *p* = 0.129, Cohen’s d = 0.206, BF_10_ = 0.443. Conversely, in the negative emotional state, the late CDA amplitudes were significantly larger in the 2T2D condition (−1.56 ± 1.04) than in either the 2T condition (−1.06 ± 1.10), t (55) = 3.001, *p* = 0.004, Cohen’s d = 0.401, BF_10_ = 7.921, or the 4T condition (−1.16 ± 1.17), t (55) = 2.014, *p* = 0.049, Cohen’s d = 0.269, BF_10_ = 0.949. However, no significant difference was detected in the late CDA amplitudes between the 2T condition and the 4T condition, t (55) = 0.620, *p* = 0.538, Cohen’s d = 0.083, BF_10_ = 0.175.

These findings revealed that the early CDA amplitude was consistently larger than the late CDA amplitude. More importantly, these results identified distinct patterns in distractor filtering between the early and late CDA components in the neutral emotional state. Specifically, the early CDA amplitude was larger in the distractor (2T2D) condition than in the low memory load (2T) condition. However, no significant difference was observed in the late CDA amplitudes between the distractor and low memory load conditions. Nevertheless, the negative emotional state showed a similar pattern for both early and late CDA components, with both amplitudes being larger in the distractor condition than in the low memory load condition.

## Discussion

While previous research has suggested that negative emotional states can enhance participants’ VWM precision but reduce the quantity of VWM (Long et al., 2020; Spachtholz et al., 2014; Xie et al., 2023), the influence of negative emotional states on distractor filtering in VWM remains poorly understood. Therefore, the aim of our study was to address this gap by exploring how negative emotional states affect the ability to filter distractors in VWM. Utilizing the CDA component, we examined whether participants could effectively exclude distractor items from VWM while in different emotional states.

In our experimental setup, we presented negative or neutral images prior to the memory array and recorded the EEG data to assess the impact of emotional states on VWM filtering. Intriguingly, we noted an inconsistent pattern between early and late CDA amplitudes in the neutral emotional block, namely, a higher early CDA amplitude in the 2T2D condition than in the 2T condition, which then equalized during the late window. This indicates that under neutral conditions, participants initially store distractor information in VWM but efficiently eliminate it during the later maintenance phase. Our results are in line with the study by Qi et al. (2014), which divided maintenance into two parts and observed the filtering effect in the late time window. Furthermore, our findings provide new refined evidence that complements previous findings of a persistent filtering effect throughout the maintenance phase (Allon & Luria, 2019; Feldmann-Wustefeld & Vogel, 2019; Liesefeld et al., 2014; Vogel et al., 2005). In our study, we introduced an additional task-irrelevant stimulus before the VWM task; therefore, we reasonably speculated that neutral stimuli, functioning as neutral emotion inducers, also would affect filtering. A neutral stimulus is likely to capture part of the attention resources and cause a deceleration of sequential distractor filtering within the VWM task. Notably, for the ERP results in the negative emotional state block, we found no significant difference in the whole CDA amplitude, early CDA amplitude, or late CDA amplitude between the 4T condition and the 2T condition. This suggests that the participants’ maximum number of maintained targets in VWM is reduced to about two items in the negative emotional state.

Another notable observation was that although the CDA results showed a reduction in the maximum number of items maintained under negative emotional states compared with neutral emotional states, the behavioral results demonstrated no significant difference in terms of the accuracy of remembering four items between the negative emotional state and neutral emotional state. Previous research has demonstrated that CDA primarily tracks the number of VWM representations (quantity) rather than the precision of VWM (He et al., 2015; Ye et al., 2014). Hence, our results could not directly reflect the impact of negative emotional states on VWM precision, as previous studies have (Xie et al., 2023). However, a plausible possibility that would explain our findings is that while the quantity of items remembered may decrease under negative emotional states, the VWM precision improves, resulting in no significant difference in the overall VWM performance for a high memory load between negative and neutral emotional states. These findings are consistent with previous research that has demonstrated that under negative emotional conditions, participants involuntarily allocate more VWM resources to a smaller number of target items, which leads to increased precision but decreased capacity for simultaneously maintaining items in VWM (Long et al., 2020; Spachtholz et al., 2014; Xie et al., 2023). The observed decrease in VWM quantity aligns with prior studies that utilized CDA to investigate the influence of negative emotional states on the representation quantity of VWM (Figueira et al., 2017, 2018). However, we did not employ direct measures of VWM precision; therefore, further research is required to confirm this possibility. Furthermore, we observed that the CDA amplitude for the 2T2D condition was significantly larger under the negative emotional state than under the neutral emotional state. These findings suggest that negative emotional states may lead participants to store more unnecessary information in the 2T2D condition than they would in the neutral emotional state. This implies that negative emotional states weaken participants’ ability to filter distractors in VWM processing, resulting in an increase in the breadth of VWM storage capacity when distractors are present. One noteworthy finding is that under a negative emotional state, the whole CDA amplitude in the 2T2D condition was not only significantly larger than the CDA amplitude in the 2T condition, but it also was marginally higher than the CDA amplitude in the 4T condition. In addition, the early CDA amplitude did not differ significantly between the 2T2D condition and the 4T condition, but the late CDA amplitude was significantly larger in the 2T2D condition than in the 4T condition. This suggests that participants stored more overall representations in the 2T2D condition than in the 4T condition, especially during the late maintenance phase. Similar instances of higher CDA amplitudes under 2T2D conditions than under 4T conditions have been found in previous studies. For instance, Qi et al. (2014) found that participants with high trait anxiety exhibited significantly higher CDA amplitudes under 2T2D conditions than under 4T conditions during the early stages of VWM maintenance. Furthermore, Ward et al. (2020) found that although a trend toward larger CDA amplitudes was evident between the 4T condition and 2T2D conditions in the safe condition (no state anxiety), the trend was reversed in the threat condition (state anxiety), with larger CDA amplitudes observed under the 2T2D condition than under the 4T condition (this trend persisted until the late stage of VWM maintenance).

The reason the participants in our study remembered more items under the 2T2D condition than under the 4T condition when in a negative emotional state may be due to entirely different VWM storage processing modes when participants are required to remember arrays that consist entirely of targets versus arrays that contain both targets and distractors. For arrays consisting only of targets, the mechanism is as follows: when participants need to remember an array consisting solely of targets, negative emotional states unconsciously enhance VWM precision, but at the cost of reducing the number of items that can be simultaneously maintained in VWM. Under the 4T condition, the CDA decreases to a level comparable to the CDA under the 2T condition in a negative emotional state block. This cost of VWM quantity might be compensated by an improvement in representation quality, which was not reflected by the CDA component but was possibly evidenced by the observation that the memory accuracy for the 4T condition was not lower in the negative emotional state block than in the neutral emotional state block. This finding aligns with previous research on the impact of negative emotional states on VWM (Long et al., 2020; Spachtholz et al., 2014; Xie et al., 2023). For arrays containing both target and distractor items, the mechanism is as follows: When participants are faced with a memory array accompanied by distractors, additional processing is required to select the target stimuli. This selection process requires the participants to encode both the target stimuli and the distractors. The negative emotional state may lead participants to involuntarily store as many items as possible, including the distractors, into VWM (even at a lower level of VWM precision). The result is a significantly higher CDA amplitude under 2T2D conditions than under either 2T or 4T conditions. This phenomenon is similar to our previous finding that participants who have limited VWM capacity involuntarily store as much distraction information as possible into VWM upon encountering potential threat distractors (even neutral faces) (Ye et al., 2018). Future research should explore this possibility by examining whether two different processing modes exist when participants need to remember arrays that consist solely of targets versus arrays that contain both targets and distractors.

Another possible explanation for the higher CDA amplitude under 2T2D conditions compared with the 4T conditions is that although CDA primarily tracks the number of items stored in VWM when the memory array consists only of targets, the modulation of the CDA amplitude under arrays containing both targets and distractors may be influenced by additional factors. Under 2T2D conditions, if the distractors are involuntarily stored in VWM, the participants may be motivated to selectively enhance the consolidation of target stimuli and exclude representations of distractors from VWM, which could lead to a larger CDA amplitude. Previous research also has suggested that the CDA amplitude is modulated by top-down attentional control (Kuo et al., 2012; Machizawa et al., 2012; Sander et al., 2011). Therefore, the presence of a higher CDA amplitude under 2T2D conditions in a negative emotional state may reflect the participants' inability to filter out distractor stimuli and the increased engagement of top-down attentional control. Considering that most previous studies on the CDA component have been conducted under paradigms in which all stimuli are targets, further research is needed to investigate the factors that modulate CDA amplitude under paradigms involving distractors. However, regardless of which explanation possibly holds, both explanations support the notion that participants automatically store more distractors in VWM under negative emotional states, indicating that negative emotional states impair participants’ ability to filter distractors.

In addition, our behavioral findings demonstrate that participants’ accuracy was significantly lower in the 4T condition than in the 2T condition, regardless of the presence of distractors. This aligns with previous research indicating that accuracy tends to decline as the number of memory items increases (Luck & Vogel, 1997). Moreover, the accuracy was slightly higher in the neutral emotional state condition than in the negative emotional state condition, particularly when remembering a low memory load (2T condition). Previous research has indicated that negative emotions can enhance participants’ VWM precision at the expense of storage capacity (Long et al., 2020; Spachtholz et al., 2014; Xie et al., 2023). However, in the context of a change detection task, the effect on overall accuracy may be greater for storage capacity than for VWM precision, especially under the condition of low memory load (i.e., the 2T condition). This may explain why previous behavioral studies have reported benefits from negative emotional states, whereas our results demonstrated a slight decrease in the accuracy performance. However, we found significant evidence for this only under the neutral emotional state condition, where the accuracy was lower in the 2T2D condition than in the 2T condition. Surprisingly, we did not observe a similar effect in the negative emotional state condition. This suggests that based on our behavioral results, the presence of distractors slightly impairs VWM storage, particularly in the context of a neutral emotional state.

Previous studies have often utilized negative emotional images to manipulate participants' emotional states, but this requires them to rate the valence of these images for deeper processing (Long et al., 2020; Souza et al., 2021; Xie & Zhang, 2016, 2017). In our study, we simply instructed our participants to passively view emotionally unrelated images, thereby minimizing the impact of target-driven, high-level processing of emotional images on subsequent VWM tasks. Furthermore, the emotional images we used (black-and-white real-world images) were entirely distinct from the stimuli used in the memory task (colored rectangles), thereby avoiding any confusion between the preceding emotional images and the memory targets.

One question worth considering is whether participants’ results under different emotional states might be influenced by their automatic storing of emotional images during image viewing. For example, when in a negative emotional state, participants might involuntarily store negative emotional images (even if the images are task irrelevant) in their VWM. This would lead to the partial occupation of VWM resources before the VWM task and would restrict the participants from fully utilizing all VWM resources in subsequent tasks. Additionally, because negative emotional images are presented centrally, the CDA cannot track the memory load associated with negative emotional images. Consequently, the results of tracking VWM quantity in the VWM task using CDA demonstrate a decrease in the maximum storage capacity of VWM for participants in a negative emotional state. However, although this hypothesis partially explains the decrease in VWM quantity under negative emotional states, it fails to account for the higher CDA amplitudes under 2T2D conditions. Therefore, we believe that the differential pattern of results between participants in negative and neutral emotional states is not solely attributable to the automatic storing of preceding emotional images but is instead the result of the differential effects of the emotional states.

A natural question arises as to whether we effectively manipulated our participants’ emotional states. Our use of the PANAS questionnaire revealed that the participants exhibited significantly lower negative emotional changes during the neutral emotional state block than during the negative emotional state block. Importantly, after the neutral emotional state block, the participants’ negative emotional change values were significantly below zero, indicating a reduction in negative emotion following exposure to the neutral emotional state block. Conversely, after the negative emotional state block, the participants’ negative emotional change values significantly exceeded zero, suggesting an increase in negative emotion following exposure to the negative emotional state block. These results demonstrate that even having participants passively view task-irrelevant emotional images allowed us to manipulate their emotional states effectively. Additionally, our results revealed distinct patterns of CDA amplitudes between participants during the neutral and negative emotional state blocks, further supporting our successful manipulation of different emotional states in the participants. However, we must note that our manipulation of the emotional state involved repeated presentation of negative versus neutral pictures before each trial, depending on the block. Although the results of the PANAS questionnaire indicated changes in emotional states, disentangling the effects of different emotional states from the immediate aftereffects of viewing the different types of images remains challenging, because these differed in valence and arousal. Consequently, when interpreting our findings, we must consider this limitation. Future research might control the method chosen for emotional arousal—such as using audio clips to induce negative emotional states, as in the study by Xie et al. (2022)—to ascertain whether the observed impairment in individuals’ distractor filtering abilities stems from the negative emotional state itself or from the passive viewing of negative stimuli.

Previous CDA studies on distractor filtering abilities have primarily focused on the filtering during the memory stimulus encoding phase (Feldmann-Wustefeld et al., 2018; Owens et al., 2012; Vogel & Machizawa, 2004). However, a recent behavioral study by Duan et al. (2023) systematically examined distractor presentation during both the encoding phase of memory stimuli and the maintenance phase following the disappearance of memory stimuli. Their findings revealed different mechanisms for filtering distractors during the encoding and maintenance phases. Future research that integrates CDA technology could explore further the differences in distractor-filtering mechanisms under different emotional states across these phases. In addition, our recent studies (Ye et al., 2018, 2023) indicated differences in filtering patterns for different emotional face distractors under neutral emotional states. Future studies could investigate whether the filtering mechanisms for different emotional face distractors are affected by different emotional states.

Our current findings significantly contribute to our understanding of the impact of negative emotional states on VWM. We have provided new evidence that negative emotional states reduce the quantity of memories stored. Moreover, our findings suggest that individuals, although able to filter out most distractors during the later stages of VWM retention under neutral emotional states, automatically store these distractors in VWM under negative emotional states. This revelation uncovers distinct patterns in how individuals handle distraction information in VWM while under different emotional states. The identification of different storage patterns in negative emotional states enhances our comprehension of how emotions influence cognitive functions, thereby providing a foundation for future investigations into emotional regulation and mental health. This new research focus can aid in the development of interventions and therapies for individuals struggling with emotional disorders or trauma-related difficulties.

### Supplementary Information

Below is the link to the electronic supplementary material.

Supplementary file1 (DOCX 755 KB)

## Data Availability

The data are available through the Open Science Framework at https://osf.io/bshm2/.
