# Supplementary material for: A negative emotional state impairs individuals’ ability to filter distractors from working memory: an ERP study
Source: Cogn Affect Behav Neurosci. 2024 Feb 13;24(3):491–504. doi: 10.3758/s13415-024-01166-z (PMC11078828; doi:10.3758/s13415-024-01166-z)
Supplement: Supplementary file 1 — Supplementary file1 (DOCX 755 KB) [file 13415_2024_1166_MOESM1_ESM.docx]

**Supplementary Materials**

**A negative emotional state impairs individuals' ability to filter out distractors from working memory: An ERP study**

Chaoxiong Ye^1,2,3^, Ruyi Liu^1,2^, Lijing Guo^2^, Guoying Zhao^3^, Qiang Liu^1,4*^

1. Institute of Brain and Psychological Sciences, Sichuan Normal University, 610068, Chengdu, China.

2. Department of Psychology, University of Jyvaskyla, 40014, Jyvaskyla, Finland.

3. Center for Machine Vision and Signal Analysis, University of Oulu, 90014, Oulu, Finland.

4. Research Center of Brain and Cognitive Neuroscience, Liaoning Normal University, 116029, Dalian, China.

**Author of correspondence**: Qiang Liu (lq780614@163.com), Institute of Brain and Psychological Sciences, Sichuan Normal University, Chengdu, 610068, China.

## Analyses of reaction times

The reaction times were computed for each condition under different emotional state blocks. We only computed the average reaction time for correctly responded trials for each condition. We analyzed the reaction time two-way repeated measures by performing ANOVA, with emotional state (neutral vs. negative) and memory condition (2T vs. 2T2D vs. 4T) as within-subject factors.

As depicted in Figure S1, the ANOVA results for reaction time revealed a significant main effect of the memory condition (mean reaction time for the 2T, 2T2D and 4T conditions: 697.23 ± 134.87, 718.45 ± 139.57, 748.00 ± 152.25, respectively), F (2,110) = 28.590, *p* < 0.001, *η_p_^2^* = 0.342, whereas no significant main effect was observed for the emotional state (mean reaction time for the neutral emotional state and negative emotional state: 712.53 ± 154.24, 729.92 ± 142.70, respectively), F (1,55) = 1.601, *p* = 0.211, *η_p_^2^* = 0.028, and no significant interaction was evident between the memory condition and emotional state, F (2,110) = 1.645, *p* = 0.198, *η_p_^2^* = 0.029.

Planned pairwise comparisons revealed that under the neutral emotional state, the reaction time was significantly longer in the 4T (755.6 ±158.4) condition than in the 2T condition (709.2 ± 137.5), t (55) = 6.407, *p* < 0.001, Cohen's d = 0.856, BF_10_ > 1000, and the 2T2D condition (725.0 ± 142.7), t (55) = 3.499, *p* < 0.001, Cohen's d = 0.468, BF_10_ = 29.182. Additionally, the reaction time was significantly longer in the 2T2D condition than in the 2T condition, t (55) = 6.254, *p* < 0.001, Cohen's d = 0.836, BF_10_ > 1000. Under the negative emotional state, the reaction time was significantly longer in the 4T condition (740.4 ± 165.0) than in the 2T condition (685.2 ± 152.6), t (55) = 4.891, p < 0.001, Cohen's d = 0.654, BF_10_ > 1000, and the 2T2D condition (711.9 ± 154.3), t (55) = 3.980, *p* < 0.001, Cohen's d = 0.532, BF_10_ = 115.969. Additionally, the reaction time was significantly shorter in the 2T condition than in the 2T2D condition, t (55) = 2.993, *p* = 0.004, Cohen's d = 0.400, BF_10_ = 7.775. Furthermore, no significant difference was observed in the reaction time for the 2T condition under the neutral and negative emotional states, t (55) = 1.665, *p* = 0.102, Cohen's d = 0.223, BF_10_ = 0.532. Similarly, no significant difference was evident in the reaction time for the 2T2D condition under the neutral and negative emotional states, t (55) = 0.952, *p* = 0.345, Cohen's d = 0.127, BF_10_ = 0.224, and no significant difference was apparent in the reaction time for the 4T condition under the neutral and negative emotional states, t (55) = 1.036, *p* = 0.305, Cohen's d = 0.138, BF_10_ = 0.243.

**Figure S1.** Reaction time results (mean and standard error of the mean) are presented separately for the neutral emotional state (left) and the negative emotional state (right) across different memory conditions.

#### Discussion

Our results indicate that the participants exhibited the shortest reaction times in the 2T condition, followed by the 2T2D condition, and the longest reaction times in the 4T condition. This result pattern is similar to those obtained for the accuracy results, suggesting that the highest accuracy and shortest reaction times occurred in the 2T condition, compared to the 2T2D condition and 4T condition, and the lowest accuracy and longest reaction times occurred in the 4T condition compared to the other conditions. These findings indicate the absence of a trade-off between accuracy and reaction time in our observed data. However, as our participants were instructed to prioritize accuracy over speed during the experiment, caution should be exercised when interpreting the reaction time results.

## Exploratory analyses of the N2pc components

The N2pc is a prominent unilateral posterior negative brain wave and is commonly interpreted as an index of the attention allocated to a target stimulus ([Eimer, 1996](#_ENREF_3); [Liu et al., 2016](#_ENREF_7); [Luck & Hillyard, 1994](#_ENREF_8); [Zhao et al., 2011](#_ENREF_14)). The latency period reflects the point at which attention is directed to the target stimulus. Similar to previous studies that utilized CDA ([Feldmann-Wustefeld & Vogel, 2019](#_ENREF_4)), we performed exploratory analyses to compare the N2pc components under different conditions. The preprocessing and calculation of the amplitudes of the difference waveforms of the N2pc components were conducted as described for the CDA component.

#### N2pc (180–230 ms)

The N2pc was measured for each memory condition (2T vs. 2T2D vs. 4T) in the neutral and negative emotional blocks as the difference in the mean amplitude between the ipsilateral and contralateral waveforms recorded at the analyzed electrodes (PO7/PO8) ([Feldmann-Wustefeld & Vogel, 2019](#_ENREF_4); [Luck & Hillyard, 1994](#_ENREF_8)) at 180–230 ms after the onset of the memory array (Figure S2).

**Figure S2.** Difference waveforms (contralateral waves minus ipsilateral waves) of the average ERPs for all memory conditions in the neutral (A) and negative (B) emotional conditions. The dashed lines indicate the analysis time window used to calculate the mean N2pc amplitude.

The N2pc amplitudes for each memory condition in the neutral and negative emotional blocks are presented in Figure S3. The ANOVA revealed a significant main effect of the memory condition, F (2,110) = 3.419, *p* = 0.036, *η_p_^2^* = 0.059, but no significant main effect of the emotional block, F (1,55) = 0.900, *p* = 0.347, *η_p_^2^* = 0.016, and no significant interaction between the memory condition and the emotion condition, F (2,110) = 0.575, *p* = 0.564, *η_p_^2^* = 0.010.

Planned pairwise comparisons indicated that in the neutral emotional state, the mean amplitude of the N2pc was significantly more negative for the 2T2D condition (-0.04 ± 1.02) than for the 2T condition (0.19 ± 0.81), t (55) = 2.124, *p* = 0.038, Cohen's d = 0.284, BF_10_ = 1.162. However, the mean N2pc amplitudes in the 4T condition (0.02 ± 0.89) did not significantly differ from those in the 2T condition, t (55) = 1.573, *p* = 0.122, Cohen's d = 0.210, BF_10_ = 0.464, or from those in the 2T2D condition, t (55) = 0.527, *p* = 0.600, Cohen's d = 0.070, BF_10_ = 0.167. Conversely, in the negative emotional state, the N2pc amplitude was marginally significantly more negative in the 2T2D condition (-0.15 ± 0.90) than in the 2T condition (0.07 ± 0.97), t (55) = 1.723, *p* = 0.090, Cohen's d = 0.230, BF_10_ = 0.582. However, the N2pc amplitudes did not significantly differ between the 4T condition (0.05 ± 0.86) and the 2T condition, t (55) = 0.115, *p* = 0.909, Cohen's d = 0.015, BF_10_= 0.147, or the 2T2D condition, t (55) = 1.518, *p* = 0.135, Cohen's d = 0.203, BF_10_ = 0.429.

The results also showed no significant differences between emotional states for the N2pc amplitudes in the 2T condition, t (55) = 1.255, *p* = 0.215, Cohen's d = 0.168, BF_10_ = 0.307, in the 2T2D condition, t (55) = 0.825, *p* = 0.413, Cohen's d = 0.110, BF_10_ = 0.202, or in the 4T condition, t (55) = 0.262, *p* = 0.794, Cohen's d = 0.035, BF_10_ = 0.151.


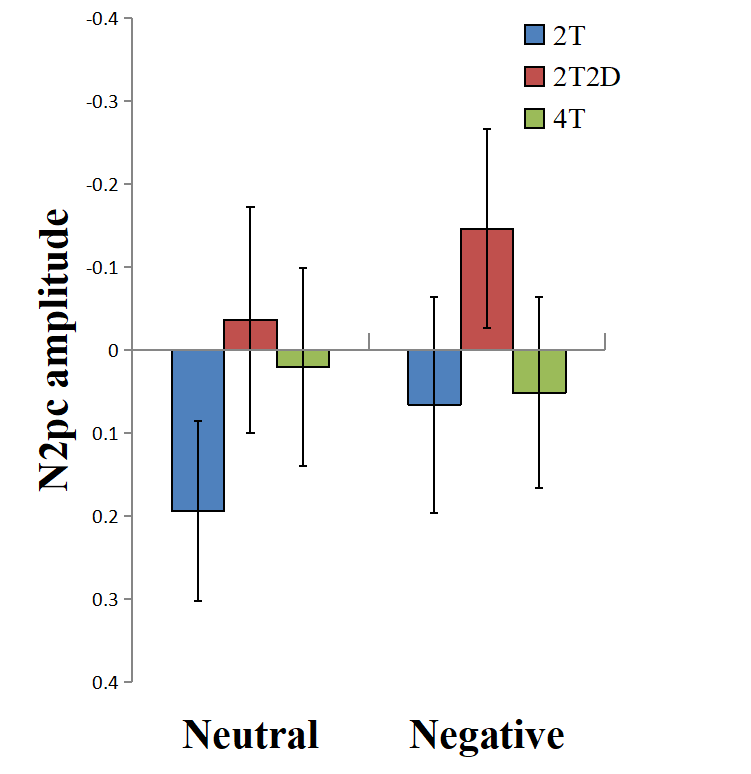


**Figure S3.** The N2pc amplitudes in the neutral (left) and negative (right) emotional state blocks for each memory condition. Error bars represent standard errors.

#### Discussion

The N2pc component provides valuable insights into an individual's allocation of attentional resources prior to encoding. Consequently, when a greater number of items need to be memorized, more attentional resources are required, resulting in an increase in the N2pc amplitude ([Eimer, 1996](#_ENREF_3); [Liu et al., 2016](#_ENREF_7)). The N2pc results in our study indicate that during the early period of VWM, the participants allocated more attentional resources to the 2T2D array than to the 2T array, irrespective of whether the emotional state was neutral or negative. Conversely, no significant difference was observed between the 2T2D condition and the 4T condition, suggesting that the participants did not distinguish between a distractor and a target item during that time. Instead, they allocated equal attentional resources to both the 2T2D and 4T arrays, which contained the same number of items.

Considering our CDA results, which showed that the participants did not effectively filter out distractors during the early time window (300–600 ms), irrespective of emotional states, we can infer that the inability to detect distractors (as indicated by early CDA) is associated with the allocation of attention to the distractors (as indicated by the N2pc).

## 3. Examining the correlation between VWM capacity and distractor filtering

Individual VWM capacity has been demonstrated to predict participants' efficacy in filtering distractors, with low-VWM-capacity participants performing worse in the filtering task compared to high-VWM-capacity participants ([Vogel et al., 2005](#_ENREF_10); [Ye et al., 2018](#_ENREF_13)). Here, we also investigated the correlation between VWM capacity and filtering ability.

Prior to the main EEG task with emotional induction, the participants need to conduct a behavioral measurement with a color change detection task to assess their individual VWM capacity.

### Methods used for the VWM capacity measurement

#### Materials

In each memory array, six squares with different colors were randomly selected from a color pool of seven colors (red: 255, 0,0; green: 0, 210, 0; blue: 0, 0, 210; orange: 228, 108, 10; yellow: 210, 210, 0; violet: 112, 48, 160; pink: 255, 75, 186) and were presented within an invisible 9.8°×7.3° rectangle centered on the fixation, against a gray background (6.1 cd/m^2^, RGB: 128, 128, 128) on a 21-inch LCD monitor (refresh rate 75 Hz) at a viewing distance of 60 cm. A single square subtended a visual angle of about 0.65° and the two squares sustained an interval of at least 1.5°. The capacity task was programmed via E-prime 2.0.

#### Procedure

At the beginning of the capacity task, the memory array consisted of six colors and was displayed for 200 ms, followed by an interval with only a fixation for 900 ms. The test array began next, and one colored square appeared at one of the six locations occupied by colors in the memory array (as in Figure S4). The participants were asked to identify whether the color in the test array was identical to the color at the same location in memory, and to press “j” if the color had changed; otherwise, they should press “f.” The color in the test array in half the trials was identical to that of the memoranda, whereas the other half changed. The test array would not disappear unless the participant pressed the keyboard or 2500 ms passed. This capacity task contained 100 trials and the total duration was about ten minutes.

**Figure S4.** An example of the procedure of VWM capacity measurement with changed test stimuli.

#### Data analyses

The VWM capacity (K) of each participant was quantified based on their results in the VWM capacity measurement. The standard formula proposed by [Cowan (2001](#_ENREF_2)) was applied: K = N × (H − F), where K is the VWM capacity, N is the size of the array (i.e., six in the present study), H is the hit rate (i.e., the proportion of correct responses when a change is present), and F is the false alarm rate (i.e., the proportion of incorrect responses when no change is present).

The filtering ability of each participant in the neutral or negative emotion state was calculated by subtracting the CDA amplitude for the 2T2D condition from the CDA amplitude for the 2T condition. A CDA difference (CDA_diff_) close to 0 represents high filtering ability, and a CDA_diff_ larger than 0 means impairment of filtering ability.

$${CDA}_{diff} = {CDA}_{2T}-{CDA}_{2T2D}$$

Two-tailed Pearson’s *r* correlation coefficients between K and CDA_diff_ were calculated for the unsegmented CDA (300–1000 ms) separately in each emotional block to investigate the relationship between VWM capacity and distractor filtering. If K values showed a significant negative correlation to CDA_diff_ in the negative emotion condition, we inferred that the individual capacity had modulated the negative emotion effect on distractor filtering. More specifically, participants with lower VWM capacity were impaired by negative emotion in the filtering task.

We also conducted a median split of the K values to divide the participants into two VWM capacity groups (high-capacity group: n = 25, mean age = 22.40 ± 3.10 years, 11 males; low-capacity group: n = 27, mean age = 21.82 ± 3.22 years, 16 males) and conducted two-way repeated measures ANOVA for CDA_diff_, with emotional state (neutral vs. negative) as the within-subject factor and VWM capacity (high vs. low) as the between-subject factor. For the follow-up comparisons of the different capacity groups’ filtering performance in each emotional state, we conducted two-tailed paired t-tests and one-sample t-tests compared to zero. If an interaction between the emotional state and the individual capacity was identified, this was considered an indication of the VWM capacity for modulation of the negative emotion effect on filtering. Furthermore, if the CDA_diff_ was larger in negative emotion trials than in neutral emotion trials for participants with low K values, but this difference disappeared for those with high K values, then a high capacity might facilitate resistance to the negative influence of the negative emotional state. However, we expected to observe a larger CDA_diff_ in the negative emotion condition than in the neutral one for the high-capacity group but a CDA_diff_ with no significant difference in the two emotion conditions for the low-capacity group if the susceptibility to negative emotion impairment was greater in the high-capacity participants than in the low-capacity participants.

A significance level of *p* < 0.05 was used for all tests. The value of η_p_^2^ was used as an estimator of the effect size for ANOVA. Cohen's d and Bayes factors were separately used as estimators of the effect size and to support the alternative hypothesis for t-tests.

### Results

#### Pearson’s r correlations

The Pearson’s *r* correlations revealed no significant association between K and CDA_diff_ in the neutral emotion condition, *r* (55) = -0.125, *p* = 0.359; or in the negative emotion condition, *r* (55) = 0.071, *p* = 0.601, as shown in Figure S5A-B.


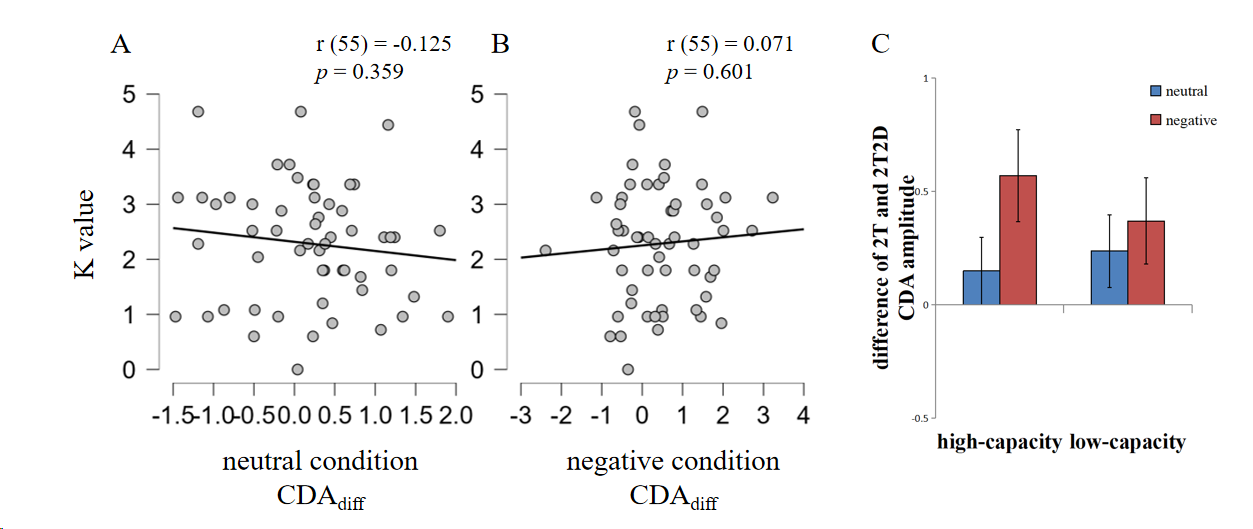


**Figure S5.** The relation between VWM capacity and distractor filtering ability. (A&B) Pearson’s r correlation results of individual VWM capacity (K) and distractor filtering ability (CDA_diff_) in neutral emotion (A) and negative emotion (B) conditions. (C) CDA amplitude differences in the 2T condition and 2T2D condition for high- or low-capacity participants with each emotional state. Error bars represent standard errors.

#### VWM capacity grouping

The independent samples t-test showed that the K values were significantly larger in the high-capacity group (3.24 ± 0.62) than in the low-capacity group (1.39 ± 0.62), t (50) = 10.689, *p* < 0.001, Cohen’s d = 2.967, BF_10_ > 1000.

#### CDA_diff_ in high- vs. low-capacity groups

The CDA_diff_ for each emotion block (neutral vs. negative) in the high-capacity or low-capacity groups is displayed in Figure S5C.

The two-way repeated measures ANOVA results indicated no significant interaction between the capacity group and emotion block, F (1,50) = 1.723, *p* = 0.195, *η_p_^2^* = 0.033, and no significant main effect of the capacity group, F (1,50) = 0.009, *p* = 0.923, *η_p_^2^* < 0.001, but the main effect of the emotion block appeared significance, F (1,50) = 4.122, *p* =0.048, *η_p_^2^* = 0.076.

Follow-up pairwise comparisons showed that among high-capacity participants, CDA_diff_ was significantly higher in the negative emotion block (0.63 ± 1.16) than in the neutral emotion block (0.01 ± 0.76), t (24) = 2.111, *p* = 0.045, Cohen’s d = 0.422, BF_10_ = 1.384. However, this significant difference did not exist in the low-capacity group (negative emotion block: 0.37 ± 1.00, neutral emotion block: 0.24 ± 0.83), t (26) = 0.576, *p* = 0.570, Cohen’s d = 0.111, BF_10_ = 0.237.

One-sample t-test analyses showed that high-capacity participants’ CDA_diff_ was significantly larger than 0 in the negative emotion block, t (24) = 2.717, *p =* 0.012, Cohen’s d = 0.543, BF_10_ = 4.093, but was not significantly different from 0 in the neutral emotion block, t (24) = 0.076, *p =* 0.940, Cohen’s d = 0.015, BF_10_ = 0.211. Within the low-capacity group, no significant difference was found between CDA_diff_ and 0 in the negative emotion block, t (26) = 1.925, *p =* 0.065, Cohen’s d = 0.371, BF_10_ = 1.011, or in the neutral emotion block, t (26) = 1.476, *p =* 0.152, Cohen’s d = 0.284, BF_10_ = 0.535.

### Discussion

We included a color change detection task to assess the participants' individual VWM capacities and we calculated CDA_diff_ to quantify their filtering abilities. These indices were utilized to investigate the relationship between VWM capacity and distractor filtering abilities in different emotional states. The differing CDA_diff_ results compared to 0 among the capacity groups demonstrated that VWM capacity modulated the impairment of negative emotion on filtering. High-capacity participants exhibited susceptibility to negative emotion in filtering tasks, as evidenced by CDA_diff_ values larger than 0 in the negative emotion block but close to 0 in the neutral emotion block. This indicates that the participants were able to reject distractors in the neutral emotion state but struggled with filtering in the negative emotion state.

Interestingly, we observed similar CDA_diff_ results with no significant difference from 0 for each emotion block among the low-capacity participants. This suggests that the low-capacity group effectively expelled distractor representations from VWM, regardless of their emotional state. Another possible explanation is that these participants reached their capacity limitations in the 2T condition, so that in the 2T2D condition, they could only memorize two stimuli without discriminating between targets and distractors. To investigate these accounts and exclude the potential influence of negative emotion on VWM capacity ([Figueira et al., 2017](#_ENREF_5); [Figueira et al., 2018](#_ENREF_6)), we compared the CDA amplitudes in each memory condition with neutral emotional induction for the low-capacity group. Paired-sample t-tests showed no significant differences in the CDA amplitudes for the 2T condition (-1.04 ± 0.93) and the 4T condition (-1.33 ± 1.02), t (26) = 1.664, p = 0.108, Cohen's d = 0.320, BF_10_ = 0.687, or for the 2T2D (-1.28 ± 0.93) and 4T conditions, t (26) = 0.284, p = 0.779, Cohen's d = 0.055, BF_10_ = 0.211. These results did not support the idea that fewer representations were being maintained in VWM during 2T2D trials than during 4T trials, suggesting that the low-capacity participants' task-relative capacities in the main task were limited to no more than 2 orientations, potentially leading to a ceiling effect in the CDA analyses.In addition, we did not find any correlations between individual capacity and filtering ability, even in the neutral emotion block, which would seem to contradict previous studies ([Vogel et al., 2005](#_ENREF_10)). An essential point to note is that our experimental protocol differed significantly from previous ones. Specifically, we presented additional task-irrelevant neutral images before the memory array, while the participants in the previous study and our study experienced a neutral emotion state. These neutral images might have captured the participants' attention based on their individual preferences or experiences, thereby interrupting the participants' filtering efficiency independently from their VWM capacity. This nonlinear impairment could have masked the correlation between capacity and filtering ability. However, the participants with a high VWM capacity generally exhibited better control of attention, leading to successful distractor filtering within the high-capacity group.

## 4. Examining the correlation between state anxiety and distractor filtering

Before the VWM capacity measurement, participants were required to complete the state anxiety section of the State-Trait Anxiety Inventory ([STAI, Spielberger et al., 1971](#_ENREF_9)), and the scores were used to investigate whether state anxiety influenced the effect of negative emotion on distractor filtering. A higher score represents a higher degree of state anxiety.

#### Data analyses

Two-tailed Pearson’s *r* correlation coefficients between state anxiety score and CDA_diff_ were calculated for the unsegmented CDA (300–1000 ms) separately in each emotional block to investigate the relationship between state anxiety and distractor filtering. If the state anxiety scores showed a significant negative correlation with CDA_diff_ in the negative emotion condition, we inferred that the state anxiety modulated the negative emotion effect on distractor filtering; more specifically, the participants who were more easily immersed in an anxiety state were impaired to a greater extent by negative emotion in the filtering task.

We also conducted a median split of the state anxiety scores to divide the participants into two state anxiety groups (high-state anxiety group: n = 28, mean age = 22.18 ± 3.19 years, 11 males; low-state anxiety group: n = 28, mean age = 22.11 ± 2.96 years, 16 males) and conducted identical analyses to those implemented when analyzing the relationship between individual capacity and distractor filtering.

### Results

#### Pearson’s r correlations

The Pearson’s *r* correlations revealed no significant association between state anxiety scores and CDA_diff_ in the neutral emotion condition, *r* (55) = -0.055, *p* = 0.688; or in the negative emotion condition, *r* (55) = 0.032, *p* = 0.813, as shown in Figure S6A-B.


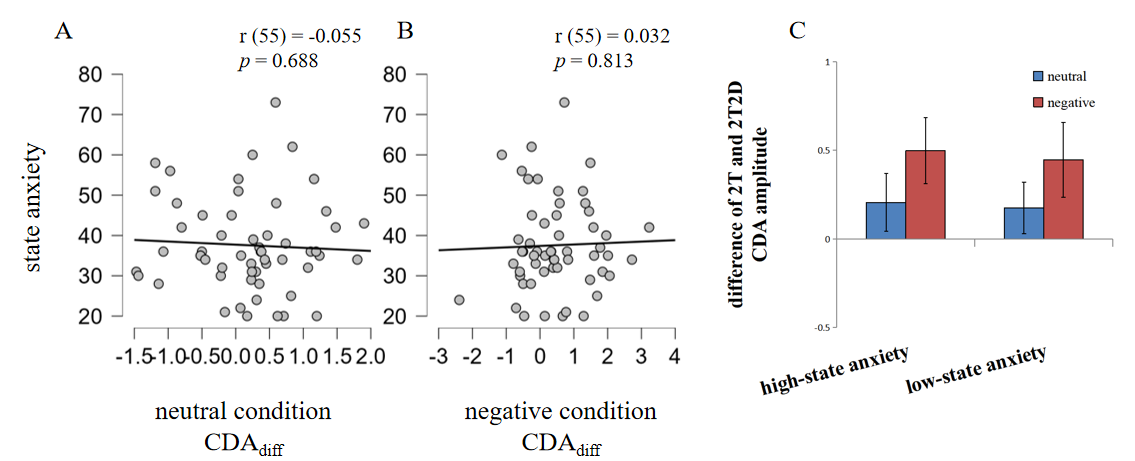


**Figure S6.** The relation between state anxiety symptoms and distractor filtering ability. (A&B) Pearson’s r correlation results of state anxiety scores and distractor filtering ability (CDA_diff_) in the neutral emotion (A) and negative emotion (B) conditions. (C) CDA amplitude differences in the 2T condition and 2T2D condition for high- or low-state anxiety participants with each emotional state. Error bars represent standard errors.

#### State anxiety grouping

The independent samples t-test showed that the state anxiety scores were significantly higher in the high-state anxiety group (46.00 ± 9.66) than in the low-state anxiety group (29.14 ± 5.40), t (54) = 8.059, *p* < 0.001, Cohen’s d = 2.154, BF_10_ > 1000.

#### CDA_diff_ in high- vs. low-state anxiety groups

The CDA_diff_ for each emotion block (neutral vs. negative) in the high-state anxiety or low-state anxiety groups is represented in Figure S6(C).

Two-way repeated measures ANOVA results indicated no significant interaction between the state anxiety group and emotion block, F (1,54) = 0.003, *p* = 0.954, *η_p_^2^* < 0.001, and no significant main effect of the state anxiety group, F (1,54) = 0.055, *p* = 0.815, *η_p_^2^* = 0.001, or of the emotion block, F (1,54) = 2.462, *p* =0.123, *η_p_^2^* = 0.044.

Follow-up pairwise comparisons showed no significant differences between CDA_diff_ in the negative emotion block (0.50 ± 0.98) or in the neutral emotion block (0.21 ± 0.86) among the high-state anxiety participants, t (27) = 1.109, *p* = 0.277, Cohen’s d = 0.210, BF_10_ = 0.350, or the low-state anxiety participants (negative emotion block: 0.45 ± 1.11, neutral emotion block: 0.18 ± 0.77), t (27) = 1.112, *p* = 0.276, Cohen’s d = 0.210, BF_10_ = 0.351.

One-sample t-test analyses showed a similarity between each state anxiety group in the result model, in which the participants’ CDA_diff_ was significantly larger than 0 in the negative emotion block (high-state anxiety group: t (27) = 2.674, *p =* 0.013, Cohen’s d = 0.505, BF_10_ = 3.804; low-state anxiety group: t (27) = 2.117, *p =* 0.044, Cohen’s d = 0.400, BF_10_ = 1.370), but was not significantly different from 0 in the neutral emotion block (high-state anxiety group: t (27) = 1.265, *p =* 0.217, Cohen’s d = 0.239, BF_10_ = 0.412; low-state anxiety group: t (27) = 1.207, *p =* 0.238, Cohen’s d = 0.228, BF_10_ = 0.386).

### Discussion

We measured the participants' state anxiety scores and investigated whether individual state anxiety influenced the impairment of distractor filtering caused by negative emotion. The results revealed no significant correlations between the state anxiety scores and CDA_diff_, or any significant interaction between the state anxiety group and the emotion block, indicating that the level of state anxiety did not modulate the negative emotion effect on VWM filtering tasks. This finding is consistent with those of [Ward et al. (2020](#_ENREF_11)), who argued that participants' anxiety state, induced by shocks, did not diminish VWM filtering ability.

Our sample participants demonstrated a normal filtering ability, as evidenced by no significant differences between CDA_diff_ and 0 in both state anxiety groups when in a neutral emotional state. In other words, the CDA amplitude did not differ between the 2T2D and 2T trials. However, as stated in our draft, regardless of the participants' state anxiety scores, CDA_diff_ was significantly larger than 0 in the negative emotion block. This implies that the negative emotion state impairs the inhibition of distractor maintenance from VWM and leads to filtering inefficiency.

## 5. Examining the correlation between trait anxiety and distractor filtering

Before the VWM capacity measurement, the participants were asked to complete the trait anxiety section of the State-Trait Anxiety Inventory ([STAI, Spielberger et al., 1971](#_ENREF_9)). The scores obtained from this section were utilized to investigate whether trait anxiety influenced the effect of negative emotion on distractor filtering. A higher score indicates a higher degree of trait anxiety.

#### Data analyses

Identical analyses were conducted to those implemented when analyzing the relationship between state anxiety and distractor filtering.

We also conducted a median split of the trait anxiety scores to divide the participants into two trait anxiety groups (high-trait anxiety group: n = 26, mean age = 22.31 ± 3.37 years, 11 males; low-trait anxiety group: n = 26, mean age = 22.23 ± 2.81 years, 14 males).

### Results

#### Pearson’s r correlations

The Pearson’s *r* correlations revealed no significant association between trait anxiety scores and CDA_diff_ in the neutral emotion condition, *r* (55) = -0.082, *p* = 0.546; or in the negative emotion condition, *r* (55) = 0.043, *p* = 0.752, as shown in Figure S7(A&B).


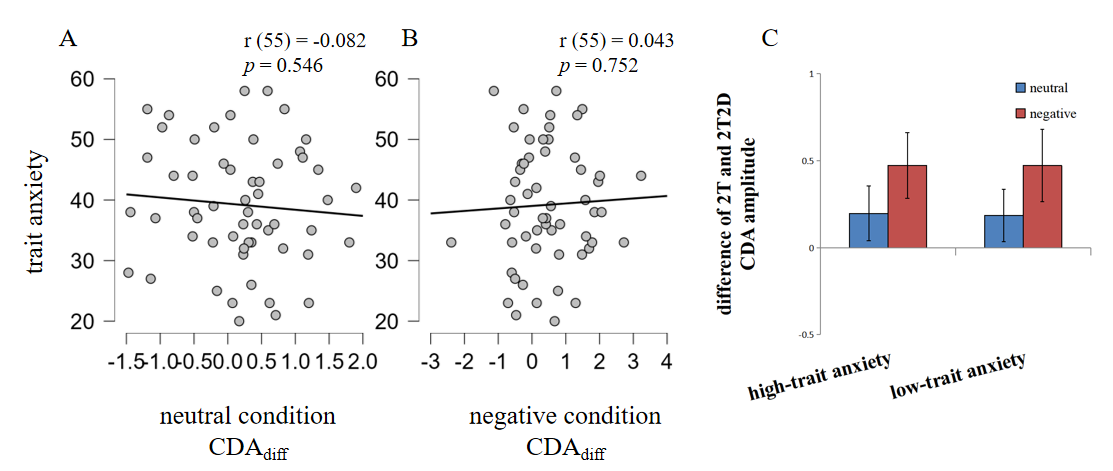


**Figure S7.** The relation between trait anxiety symptoms and distractor filtering ability. (A&B) Pearson’s r correlation results of trait anxiety scores and distractor filtering ability (CDA_diff_) in the neutral emotion (A) and negative emotion (B) conditions. (C) CDA amplitude differences in the 2T condition and 2T2D condition for high- or low-trait anxiety participants with each emotional state. Error bars represent standard errors.

#### Trait anxiety grouping

The independent samples t-test showed that trait anxiety scores were significantly larger in the high-trait anxiety group (48.04 ± 5.43) than in the low-trait anxiety group (30.54 ± 5.32), t (50) = 11.735, *p* < 0.001, Cohen’s d = 3.255, BF_10_ > 1000.

#### CDA_diff_ in high- vs. low- trait anxiety groups

The CDA_diff_ for each emotion block (neutral vs. negative) in the high-trait anxiety or low-trait anxiety groups is depicted in Figure S7(C).

The two-way repeated measures ANOVA results indicated no significant interaction between the trait anxiety group and emotion block, F (1,50) = 0.185, *p* = 0.669, *η_p_^2^* = 0.004, and no significant main effect of the trait anxiety group, F (1,50) = 0.160, *p* = 0.691, *η_p_^2^* = 0.003, or of the emotion block, F (1,50) = 1.134, *p* =0.292, *η_p_^2^* = 0.022.

Follow-up pairwise comparisons showed no significant differences between CDA_diff_ in the negative emotion block and in the neutral emotion block among the high-trait anxiety participants (negative emotion block: 0.51 ± 1.02, neutral emotion block: 0.24 ± 0.85), t (25) = 0.919, *p* = 0.367, Cohen’s d = 0.180, BF_10_ = 0.304, or the low-trait anxiety participants (negative emotion block: 0.36 ± 1.06, neutral emotion block: 0.24 ± 0.76), t (25) = 0.546, *p* = 0.590, Cohen’s d = 0.107, BF_10_ = 0.238.

One-sample t-test analyses showed the similarity between each trait anxiety group in the result model, in which the participants’ CDA_diff_ was significantly or marginally significantly larger than 0 in the negative emotion block (high-trait anxiety group: t (25) = 2.538, *p =* 0.018, Cohen’s d = 0.498, BF_10_ = 2.920; low-trait anxiety group: t (25) = 1.722, *p =* 0.097, Cohen’s d = 0.338, BF_10_ = 0.754), but was not significantly different from 0 in the neutral emotion block (high-trait anxiety group: t (25) = 1.433, *p =* 0.164, Cohen’s d = 0.281, BF_10_ = 0.514; low-trait anxiety group: t (25) = 1.629, *p =* 0.116, Cohen’s d = 0.319, BF_10_ = 0.662).

### Discussion

We assessed the participants' trait anxiety scores and investigated the potential influence of individual trait anxiety on the detrimental effects of the negative emotional state on distractor filtering. The results revealed no significant correlations between trait anxiety scores and CDA_diff_, nor did we find a significant interaction between the trait anxiety group and the emotional state block. These findings suggest that the level of trait anxiety did not modulate the impact of negative emotions on filtering in our EEG tasks.

Consistent with our findings from other analyses, CDA_diff_ was significantly greater than 0 only in the negative emotional state block, irrespective of the participants' trait anxiety scores. This finding again indicates that the presence of negative emotion impairs participants' ability to filter out distractors.

## 6. Examining the correlation between depression and distractor filtering

Prior to the VWM capacity measurement, the participants were instructed to fill out the Beck Depression Inventory II ([BDI-II, Beck et al., 1996](#_ENREF_1)). The obtained scores were utilized to examine the potential influence of depression on the impact of negative emotions on distractor filtering. A higher score on the inventory indicates a higher level of depression.

#### Data analyses

Identical analyses were conducted to those implemented when analyzing the relationship between state anxiety and distractor filtering.

We also conducted a median split of the depression scores to divide the participants into two depression groups (high-depression group: n = 28, mean age = 21.25 ± 2.73 years, 12 males; low-depression group: n = 28, mean age = 23.04 ± 3.13 years, 15 males).

### Results

#### Pearson’s r correlations

The Pearson’s *r* correlations revealed no significant association between depression scores and CDA_diff_ in the neutral emotion condition, *r* (55) = -0.036, *p* = 0.792; or in the negative emotion condition, *r* (55) = 0.040, *p* = 0.770, as shown in Figure S8(A&B).


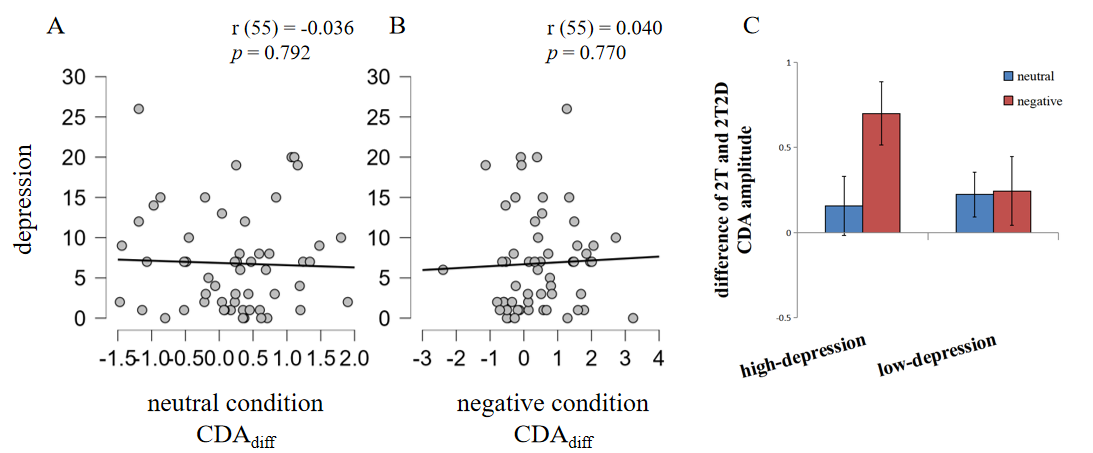


**Figure S8.** The relation between depression symptoms and distractor filtering ability. (A&B) Pearson’s r correlation results of the depression scores and distractor filtering ability (CDA_diff_) in the neutral emotion (A) and negative emotion (B) conditions. (C) CDA amplitude differences in the 2T condition and 2T2D condition for high- or low-depression participants with each emotional state. Error bars represent standard errors.

#### Depression grouping

The independent samples t-test showed that depression scores were significantly larger in the high-depression group (11.61 ± 5.27) than in the low-depression group (2.00 ± 1.74), t (54) = 9.165, *p* < 0.001, Cohen’s d = 2.449, BF_10_ > 1000.

#### CDA_diff_ in high- vs. low-depression groups

The CDA_diff_ for each emotion block (neutral vs. negative) in the high-depression or low-depression groups is presented in Figure S8(C).

Two-way repeated measures ANOVA results indicated no significant interaction between the depression group and emotion block, F (1,54) = 2.198, *p* = 0.144, *η_p_^2^* = 0.039, and no significant main effect of the depression group, F (1,54) = 1.250, *p* = 0.268, *η_p_^2^* = 0.023, or of the emotion block, F (1,54) = 2.562, *p* =0.115, *η_p_^2^* = 0.045.

Follow-up pairwise comparisons showed that among the high-depression participants, CDA_diff_ was significantly higher in the negative emotion block (0.70 ± 0.98) than in the neutral emotion block (0.16 ± 0.92), t (27) = 2.111, *p* = 0.044, Cohen’s d = 0.399, BF_10_ = 1.355. However, this difference did not exist in the low-depression group (negative emotion block: 0.24 ± 1.07, neutral emotion block: 0.22 ± 0.69), t (27) = 0.086, *p* = 0.932, Cohen’s d = 0.016, BF_10_ = 0.201.

One-sample t-test analyses showed that the high-depression participants’ CDA_diff_ was significantly larger than 0 in the negative emotion block, t (27) = 3.769 *p* < 0.001, Cohen’s d = 0.712, BF_10_ = 40.522, but was not significantly different from 0 in the neutral emotion block, t (27) = 0.905, *p =* 0.373, Cohen’s d = 0.171, BF_10_ = 0.291. Within the low-depression group, no significant difference was found between CDA_diff_ and 0 in the negative emotion block, t (27) = 1.209, *p =* 0.237, Cohen’s d = 0.228, BF_10_ = 0.387, or in the neutral emotion block, t (27) = 1.704, *p =* 0.100, Cohen’s d = 0.322, BF_10_ = 0.719.

### Discussion

We assessed the participants' depression scores and investigated whether individual levels of depression influenced the impairment of distractor filtering caused by negative emotions. Interestingly, different patterns of CDA_diff_ were observed in each emotion block between the depression groups. Specifically, among the high-depression participants, CDA_diff_ was significantly larger in the negative emotional state block than in the neutral block. However, this difference disappeared within the low-depression group. Despite the absence of significant correlations between the depression scores and CDA_diff_, as well as no significant interaction between the depression group and the emotion block, these findings suggested that individual levels of depression might regulate the impact of negative emotions on VWM filtering.Furthermore, through one-sample t-tests, we found that CDA_diff_ was higher than 0 only among the high-depression participants in the negative emotional state. This suggests that individuals with higher levels of depression were more susceptible to the impairment caused by negative emotions in the EEG task. By contrast, the low-depression participants demonstrated effective distractor filtering regardless of their emotional state, indicating that a low level of depression facilitated resistance to the negative influence of emotions on distractor filtering.

## 7. Examining the correlation between negative induction and distractor filtering

We conducted correlation analyses on the changes in the negative emotion scores ([PANAS, Watson et al., 1988](#_ENREF_12)) after a negative emotion block and CDA_diff_ implemented when analyzing the relationship between state anxiety and distractor filtering to examine whether the negative induction level adjusted the negative emotion influence on filtering. For this particular investigation, we utilized data only from the negative emotion block, and we also employed independent samples t-tests to compare CDA_diff_ among the different negative induction groups.

We also conducted a median split to divide the participants into two negative induction groups (high-negative induction group: n = 24, mean age = 22.54 ± 3.16 years, 9 males; low-negative induction group: n = 25, mean age = 21.92 ± 3.25 years, 17 males), depending on their differences in negative emotion scores after a negative emotion block.

### Results

#### Pearson’s r correlations

The Pearson’s *r* correlations revealed no significant association between negative induction level and CDA_diff_ in the negative emotion condition, *r* (55) = 0.036, *p* = 0.794, as shown in Figure S9(A).


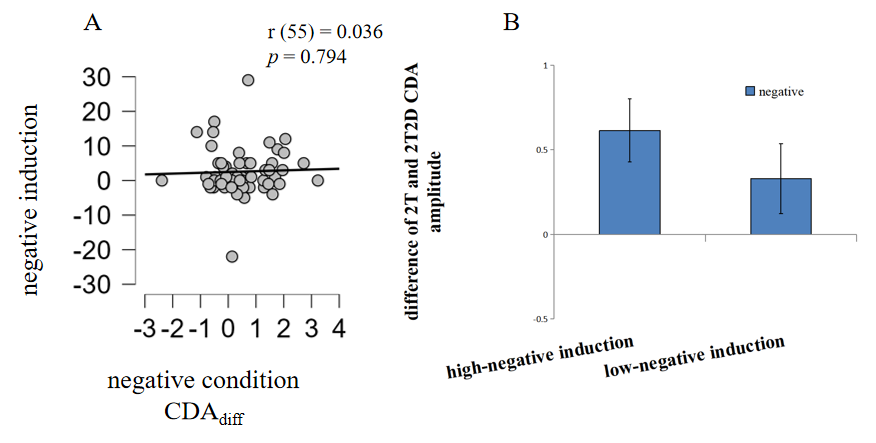


**Figure S9.** The relation between the negative induction level and distractor filtering ability. (A) Pearson’s r correlation results of the negative induction level and distractor filtering ability (CDA_diff_) in the negative emotion conditions. (C) CDA amplitude differences in the 2T condition and 2T2D condition for high- or low-negative induction participants with a negative emotional state. Error bars represent standard errors.

#### Negative induction grouping

The independent samples t-test showed that the negative induction level was significantly larger in the high-negative induction group (7.96 ± 6.04) than in the low-negative induction group (-2.16 ± 4.37), t (47) = 6.742, *p* < 0.001, Cohen’s d = 1.927, BF_10_ > 1000.

#### CDA_diff_ in high- vs. low-negative induction groups

The CDA_diff_ for the negative emotion block in the high-negative induction or low-negative induction groups is presented in Figure S9(B).

The independent samples t-test results indicated no significant differences between the negative induction groups for CDA_diff_ (high-negative induction group: 0.65 ± 1.06; low-negative induction group: 0.35 ± 1.09), t (47) = 0.977, *p* = 0.334, Cohen’s d = 0.279, BF_10_ = 0.421.

The one-sample t-test analyses showed that the high-negative induction participants’ CDA_diff_ was significantly larger than 0 in the negative emotion block, t (23) = 3.018, *p =* 0.006, Cohen’s d = 0.616, BF_10_ = 7.324. However, within the low-negative induction group, no significant difference was found between CDA_diff_ and 0 in the negative emotion block, t (24) = 1.612, *p =* 0.120, Cohen’s d = 0.322, BF_10_ = 0.655.

### Discussion

We investigated whether the participants' level of negative induction influenced the impact of negative emotion on distractor filtering during the negative emotion block. Our analysis revealed distinct differences in CDA_diff_ compared to 0 across the negative induction groups. Specifically, among the participants with a high-negative induction, the CDA amplitudes were significantly higher in 2T2D trials than in 2T trials. However, within the low-negative induction group, this difference appeared to be absent, despite the lack of a significant difference in CDA_diff_ between the two negative induction groups. These findings suggest that negative induction levels may modulate the effect of negative emotions on VWM filtering. A higher level of negative emotional state appears to correspond to a more pronounced reduction in filtering ability. However, the relationship between negative emotion intensity and its impact on filtering impairment may not follow a linear pattern, as we did not find any significant correlation between the negative induction level and CDA_diff_. Note, however, that these results indicate a complex relationship between negative emotions, induction levels, and distractor filtering, and therefore warrant further investigation for a fully comprehensive understanding.

## Reference

Beck, A. T., Steer, R. A., & Brown, G. K. (1996). Beck depression inventory-II. *Psychological Assessment*. https://doi.org/10.1037/t00742-000

Cowan, N. (2001). The magical number 4 in short-term memory: A reconsideration of mental storage capacity. *Behav Brain Sci*, *24*(1), 87-185. https://doi.org/10.1017/S0140525X01003922

Eimer, M. (1996). The N2pc component as an indicator of attentional selectivity. *Electroencephalogr Clin Neurophysiol*, *99*(3), 225-234. https://doi.org/10.1016/0013-4694(96)95711-9

Feldmann-Wustefeld, T., & Vogel, E. K. (2019). Neural Evidence for the Contribution of Active Suppression During Working Memory Filtering. *Cereb Cortex*, *29*(2), 529-543. https://doi.org/10.1093/cercor/bhx336

Figueira, J. S. B., Oliveira, L., Pereira, M. G., Pacheco, L. B., Lobo, I., Motta-Ribeiro, G. C., & David, I. A. (2017). An unpleasant emotional state reduces working memory capacity: electrophysiological evidence. *Soc Cogn Affect Neurosci*, *12*(6), 984-992. https://doi.org/10.1093/scan/nsx030

Figueira, J. S. B., Pacheco, L. B., Lobo, I., Volchan, E., Pereira, M. G., de Oliveira, L., & David, I. A. (2018). "Keep That in Mind!" The Role of Positive Affect in Working Memory for Maintaining Goal-Relevant Information. *Front Psychol*, *9*, 1228. https://doi.org/10.3389/fpsyg.2018.01228

Liu, Q., Lin, S., Zhao, G., & Roberson, D. (2016). The effect of modulating top-down attention deployment on the N2pc/PCN. *Biol Psychol*, *117*, 187-193. https://doi.org/10.1016/j.biopsycho.2016.04.004

Luck, S. J., & Hillyard, S. A. (1994). Spatial filtering during visual search: evidence from human electrophysiology. *J Exp Psychol Hum Percept Perform*, *20*(5), 1000-1014. https://doi.org/10.1037//0096-1523.20.5.1000

Spielberger, C. D., Gonzalez-Reigosa, F., Martinez-Urrutia, A., Natalicio, L. F., & Natalicio, D. S. (1971). The state-trait anxiety inventory. *Revista Interamericana de Psicologia/Interamerican journal of psychology*, *5*(3 & 4). https://doi.org/10.30849/rip/ijp.v5i3%20&%204.620

Vogel, E. K., McCollough, A. W., & Machizawa, M. G. (2005). Neural measures reveal individual differences in controlling access to working memory. *Nature*, *438*, 500-503. https://doi.org/10.1038/nature04171

Ward, R. T., Lotfi, S., Sallmann, H., Lee, H. J., & Larson, C. L. (2020). State anxiety reduces working memory capacity but does not impact filtering cost for neutral distracters. *Psychophysiology*, e13625. https://doi.org/10.1111/psyp.13625

Watson, D., Clark, L. A., & Tellegen, A. (1988). Development and validation of brief measures of positive and negative affect: the PANAS scales. *J Pers Soc Psychol*, *54*(6), 1063. https://doi.org/10.1037/0022-3514.54.6.1063

Ye, C., Xu, Q., Liu, Q., Cong, F., Saariluoma, P., Ristaniemi, T., & Astikainen, P. (2018). The impact of visual working memory capacity on the filtering efficiency of emotional face distractors. *Biol Psychol*, *138*, 63-72. https://doi.org/10.1016/j.biopsycho.2018.08.009

Zhao, G., Liu, Q., Zhang, Y., Jiao, J., Zhang, Q., Sun, H., & Li, H. (2011). The amplitude of N2pc reflects the physical disparity between target item and distracters. *Neurosci Lett*, *491*(1), 68-72. https://doi.org/10.1016/j.neulet.2010.12.066
